# Protective Role of Andrographolide in Bleomycin-Induced Pulmonary Fibrosis in Mice

**DOI:** 10.3390/ijms141223581

**Published:** 2013-12-03

**Authors:** Tao Zhu, Wei Zhang, Min Xiao, Hongying Chen, Hong Jin

**Affiliations:** 1 Division of Pulmonary and Critical Care Medicine, Department of Internal Medicine, and State Key Laboratory of Biotherapy of China, West China Hospital of Sichuan University, Chengdu 610041, Sichuan, China; E-Mails: zhutao15452@163.com (T.Z.); xiaomin_wcms@163.com (M.X.); 2 Respiratory Medicine, First Affiliated Hospital of Chengdu Medical College, Chengdu 610500, Sichuan, China; E-Mail: zhangweicdmc@126.com; 3 Laboratory of Cellular and Molecular Biology, West China Hospital of Sichuan University, Chengdu 610041, Sichuan, China; E-Mail: chenghongyingwcms@126.com

**Keywords:** andrographolide, bleomycin (BLM), pulmonary fibrosis, transforming growth factor-β1 (TGF-β1), alpha-smooth muscle actin (α-SMA), nuclear factor-κB (NF-κB)

## Abstract

Idiopathic pulmonary fibrosis (IPF) is a chronic devastating disease with poor prognosis. Multiple pathological processes, including inflammation, epithelial mesenchymal transition (EMT), apoptosis, and oxidative stress, are involved in the pathogenesis of IPF. Recent findings suggested that nuclear factor-κB (NF-κB) is constitutively activated in IPF and acts as a central regulator in the pathogenesis of IPF. The aim of our study was to reveal the value of andrographolide on bleomycin-induced inflammation and fibrosis in mice. The indicated dosages of andrographolide were administered in mice with bleomycin-induced pulmonary fibrosis. On day 21, cell counts of total cells, macrophages, neutrophils and lymphocytes, alone with TNF-α in bronchoalveolar lavage fluid (BALF) were measured. HE staining and Masson’s trichrome (MT) staining were used to observe the histological alterations of lungs. The Ashcroft score and hydroxyproline content of lungs were also measured. TGF-β1 and α-SMA mRNA and protein were analyzed. Activation of NF-κB was determined by western blotting and electrophoretic mobility shift assay (EMSA). On day 21 after bleomycin stimulation, andrographolide dose-dependently inhibited the inflammatory cells and TNF-α in BALF. Meanwhile, our data demonstrated that the Ashcroft score and hydroxyproline content of the bleomycin-stimulated lung were reduced by andrographolide administration. Furthermore, andrographloide suppressed TGF-β1 and α-SMA mRNA and protein expression in bleomycin-induced pulmonary fibrosis. Meanwhile, andrographolide significantly dose-dependently inhibited the ratio of phospho-NF-κB p65/total NF-κB p65 and NF-κB p65 DNA binding activities. Our findings indicate that andrographolide compromised bleomycin-induced pulmonary inflammation and fibrosis possibly through inactivation of NF-κB. Andrographolide holds promise as a novel drug to treat the devastating disease of pulmonary fibrosis.

## 1. Introduction

Although significant progress has been made in understanding the molecular mechanisms of the pathogenesis of idiopathic pulmonary fibrosis (IPF), the treatment is still limited, and the prognosis is still not optimistic [1–3]. According to clinical studies, the value of corticosteroids in IPF proved disappointing [4,5] and reports showed that the five-year survival rate of IPF is less than 50% [1–3,5]. Therefore, new therapeutic drugs for this unmet medical need are of particular interest.

IPF is a lethal, interstitial lung disorder that features persistent and chronic tissue scarring. The studies found that inflammation, apoptosis, oxidative stress, and epithelial mesenchymal transition (EMT) were involved in the pathogenesis of IPF [5–10]. Studies have indicated that the nuclear factor-κB (NF-κB) transcription factor is considered a central regulator of inflammation [11,12]. Meanwhile, some studies also demonstrated that over activation of NF-κB was crucial in the IPF-associated inflammatory process [13,14]. Bleomycin (BLM)-induced mouse pulmonary fibrosis is a commonly used model in IPF research [6,15]; *in vivo* studies showed that a suppressed or down-regulated NF-κB signaling pathway could attenuate BLM-induced pulmonary fibrosis [13–18], while *in vitro* studies also found that the NF-κB signaling pathway contributed substantially to the regulation of TGF-β, a critical inflammatory mediator in myofibroblast proliferation and EMT [13–18].

*Andrographis panicula* is a traditional herb widely found and used as folk medicine in China and India for the treatment of diarrhoea, dysentery, viral infection and fever. Andrographolide, an active component extracted and purified from *Andrographis panicula*, is currently prescribed for treatment of inflammatory associated diseases, including asthma, laryngitis, upper respiratory tract infection and rheumatoid arthritis in China and southeast Asia countries [11,19–21]. Recently, several studies have showed that the anti-inflammatory properties of andrographolide result from the inactivation of NF-κB [11,19–21]. Additionally, a clinical study found that rheumatoid arthritis symptoms were relieved after andrographolide treatment [22]. Our previous data also indicated that andrographolide dose-dependently suppressed the severity of LPS-induced acute lung injury (ALI), possibly by means of andrographolide-mediated NF-κB inhibition at the level of IKKβ activation [11,20].

Therefore, the purpose of this study was to investigate whether andrographolide could attenuate BLM-induced pulmonary fibrosis in a mouse model of IPF via inactivation of NF-κB.

## 2. Results

### 2.1. Andrographolide Reduces Cell Counts and TNF-α in BALF in BLM-Induced Pulmonary Fibrosis in Mice

**Figure 1.**
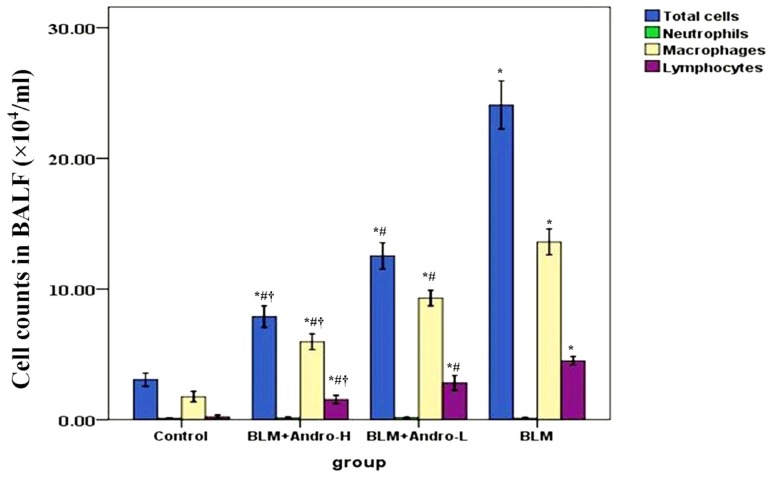
Andrographolide reduces cell counts in BALF in BLM induced pulmonary fibrosis. Twenty-one days after BLM injection (3 mg/kg) with or without andrographolide treatments (1 mg/kg, BLM + Andro-L group or 10 mg/kg, BLM + Andro-H group), mice were exsanguinated and their lungs were lavaged. Cells in the BALF were collected and cytospin preparations were made. Total cells, neutrophils, macrophages and lymphocytes in BALF were analyzed. Each bar represents the mean ± SD of 10 mice. *p < 0.01 compared with Control; ^#^*p* < 0.01 compared with BLM; ^†^*p* < 0.01 compared with BLM + Andro-L.

**Figure 2.**
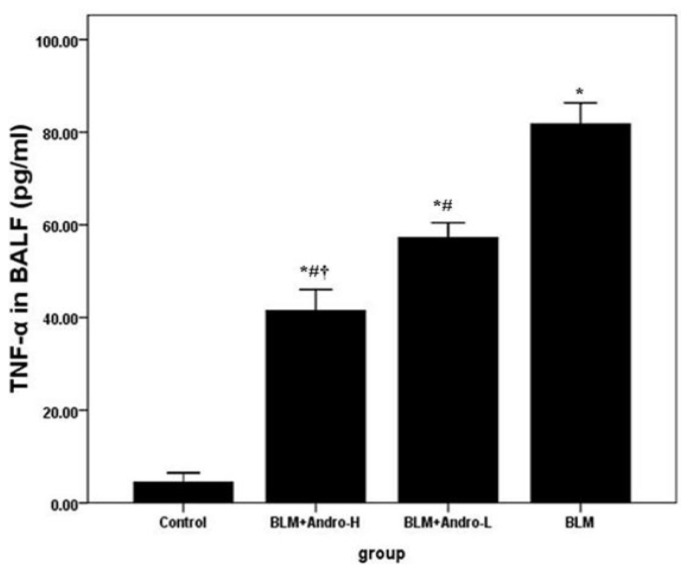
Andrographolide down-regulates TNF-α in BALF. Twenty-one days after LPS injection with or without andrographolide treatments, mice were sacrificed, their lungs were lavaged and the BALF were collected. TNF-α was detected by ELISA. Each bar represents the mean ± SD of 10 mice. *p < 0.01 compared with Control; ^#^*p* < 0.01 compared with BLM; ^†^*p* < 0.01 compared with BLM + Andro-L.

To analyze the severity of inflammation in lung, cell counts and the content of TNF-α in BALF were measured in our study. Our data showed that the number of total cells, macrophages and lymphocytes, and the level of TNF-α in BALF were significantly increased on day 21 after BLM injection. Andrographolide administrations dose-dependently reduced the numbers of total cells, macrophages and lymphocytes, and the level of TNF-α in BALF in BLM-stimulated mice (Figures 1 and 2). Meanwhile, no difference was found on the number of neutrophils in BALF among different groups (Figure 1).

### 2.2. Andrographolide Attenuates BLM Induced Pulmonary Fibrosis in Mice

**Figure 3.**
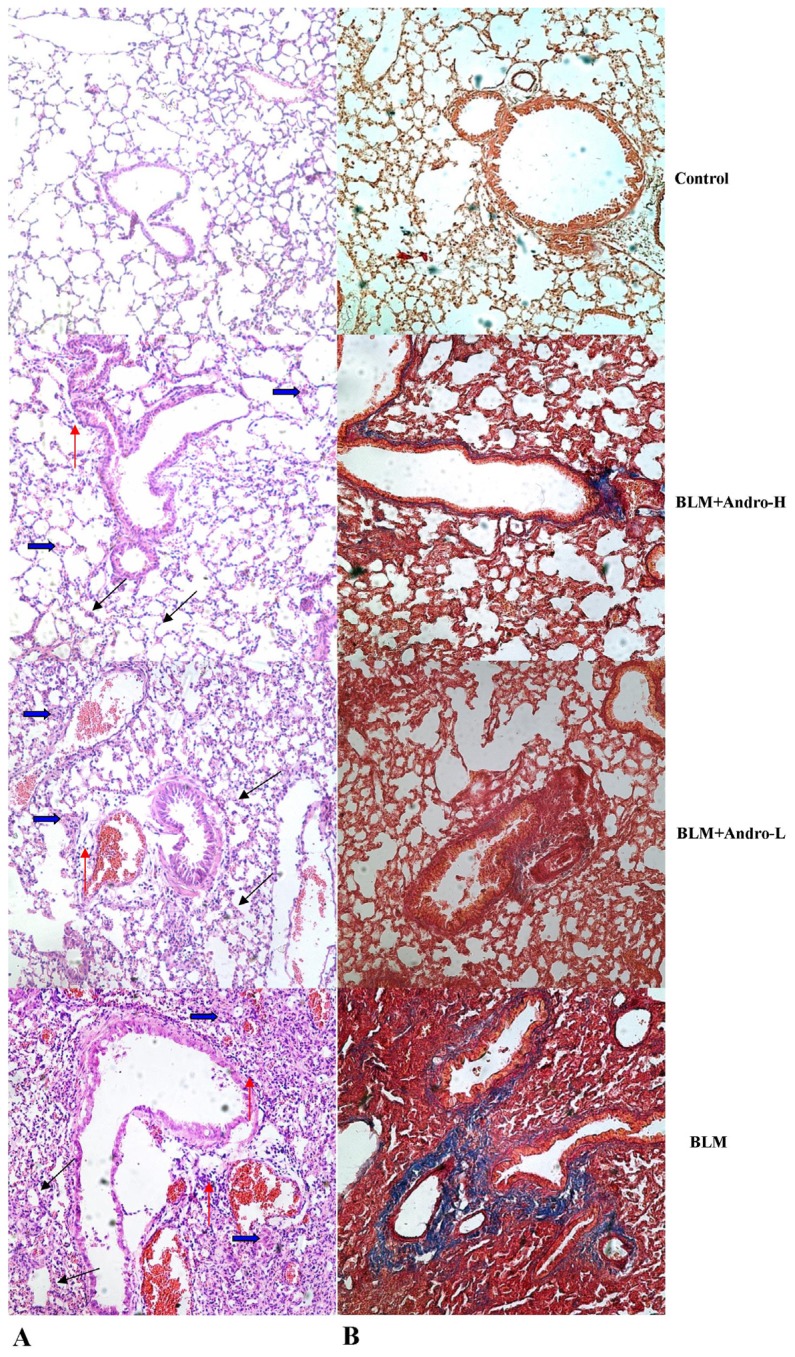

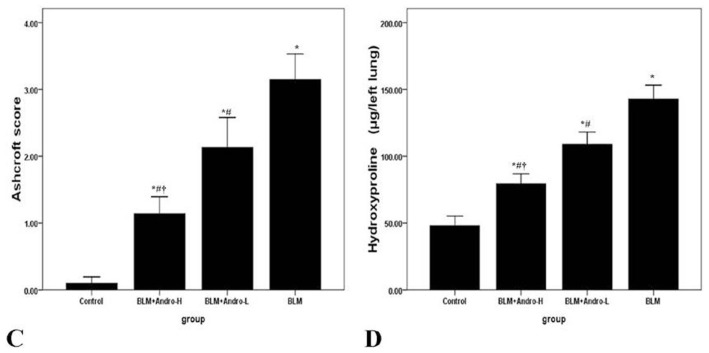
Andrographolide attenuates BLM-induced pulmonary fibrosis in mice. Twenty-one days after BLM injection with or without andrographolide treatments, mice were exsanguinated and their lungs were removed. Then, right lower lung tissue sections were stained with hematoxylin and eosin (H & E) and Masson’s trichrome (MT) staining. The hydroxyproline content of left lungs were measured. (**A**) The figure demonstrates a representative view (HE staining ×400) from each group; (**B**) The figure demonstrates a representative view (MT staining ×400) from each group; (**C**) Degree of lung fibrosis was measured with the Ashcroft score system; (**D**) Degree of collagen deposition in lung was measured with hydroxyproline content. Each bar represents the mean ± SD of 10 mice. *p < 0.01 compared with Control; ^#^*p* < 0.01 compared with BLM; ^†^*p* < 0.01 compared with BLM + Andro-L. (Thickened alveolar wall (black arrow); Deposition of mature collagen in the interstitium (blue arrow); Infiltration of inflammatory cells (orange arrow)).

To observe the pathological changes in lung, HE staining and MT staining were performed. Then, the histological evaluation of lung sections 21 days after the BLM injection revealed evidence of obvious alveolar wall thickening, massive infiltration of inflammatory cells, and excessive deposition of mature collagen in the interstitium. However, after andrographolide treatments, the pathological changes in the lung tissues were relieved. The mice in the control group showed no histological changes. As for pulmonary fibrosis evaluation, hydroxyproline content was measured. On day 21, the mice received BLM injection showed more hydroxyproline content than in control. Nevertheless, hydroxyproline content significantly reduced after andrographolide administrations (Figure 3). Additionally, the effect of andrographolide in pulmonary fibrosis was in a dose-dependent manner.

### 2.3. Andrographolide Inhibits TGF-β1 and α-SMA Expression in BLM-Induced Pulmonary Fibrosis in Mice

**Figure 4.**
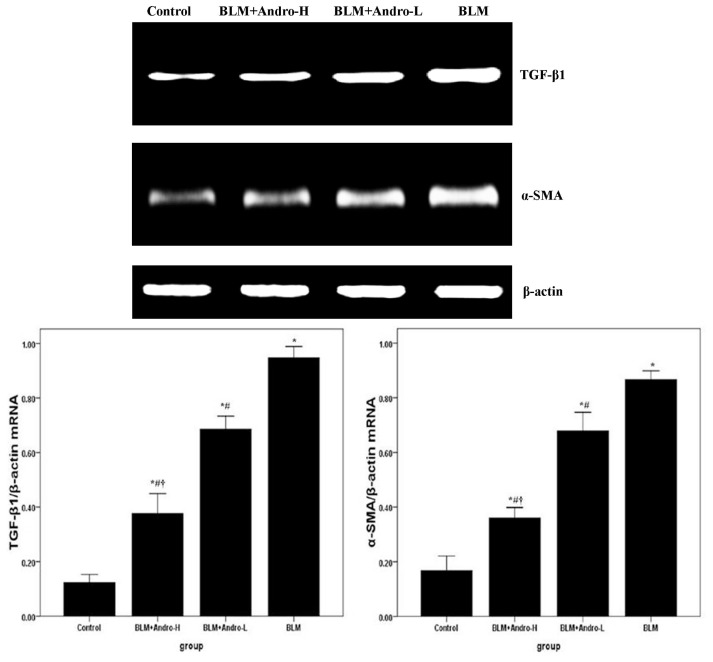
Andrographolide reduces TGF-β1 and α-SMA mRNA in lung. Twenty-one days after BLM injection with or without andrographolide treatments, mice were exsanguinated and their lungs were removed. RT-PCR was performed to detect TGF-β1 and α-SMA mRNA expression in the lung tissues. TGF-β1 and α-SMA mRNA level in each sample was expressed as a ratio of TGF-β1 or α-SMA gray value to β-actin. Each bar represents the mean ± SD of 10 mice. *p < 0.01 compared with Control; ^#^*p* < 0.01 compared with BLM; ^†^*p* < 0.01 compared with BLM + Andro-L.

**Figure 5.**
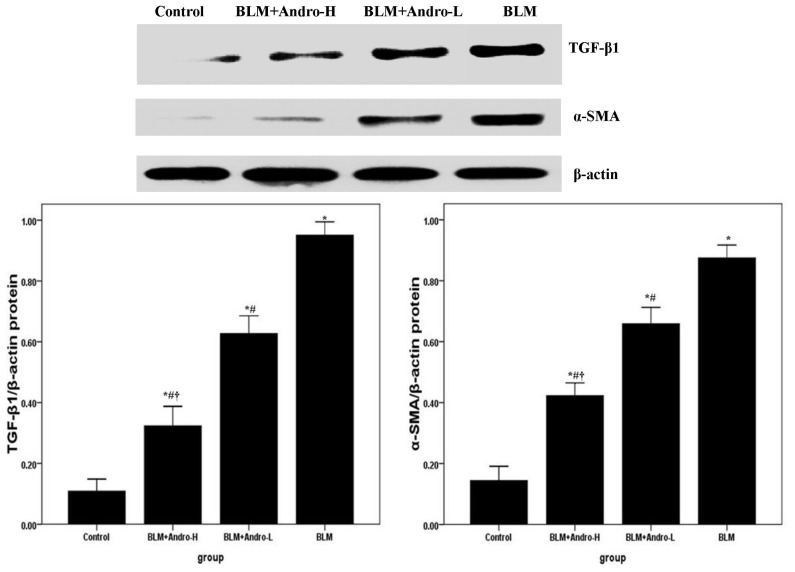
Andrographolide reduces TGF-β1 and α-SMA protein in lung. Twenty-one days after BLM injection with or without andrographolide treatments, mice were sacrificed and their lungs were removed. Western blotting was performed to detect TGF-β1 and α-SMA protein expression in the lung tissues. TGF-β1 and α-SMA protein level in each sample was expressed as a ratio of TGF-β1 or α-SMA gray value to β-actin. Each bar represents the mean ± SD of 10 mice. *p < 0.01 compared with Control; ^#^*p* < 0.01 compared with BLM; ^†^*p* < 0.01 compared with BLM + Andro-L.

TGF-β1 and its downstream molecule α-SMA were known to be critical for pulmonary fibrosis [6,7,17,18]. Compared with the control group, enhanced mRNA and protein expression of TGF-β1 and α-SMA was found on day 21 after BLM injection (Figures 4 and 5). Meanwhile, Figures 4 and 5 also showed that BLM-stimulated enhanced TGF-β1 and α-SMA expression was dose-dependently inhibited by andrographolide administrations.

### 2.4. Andrographolide Inhibits NF-κB p65 Activation and DNA Binding Activity in BLM-Induced Pulmonary Fibrosis in Mice

**Figure 6.**
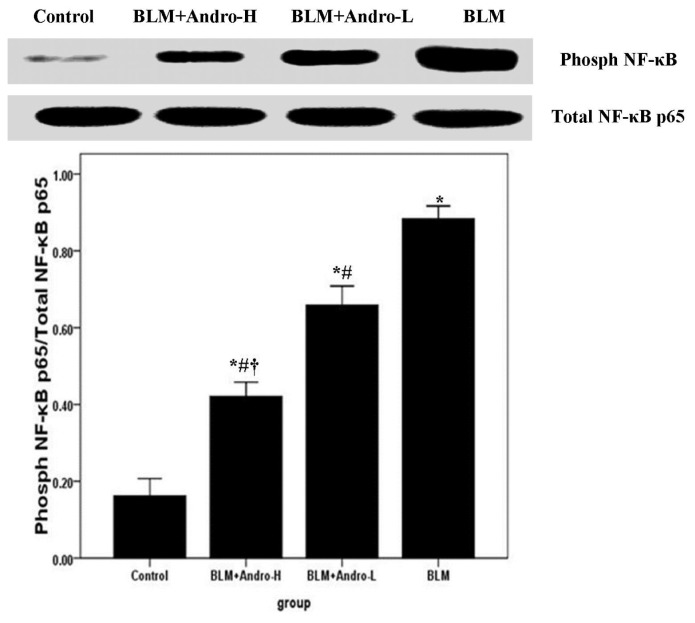
Andrographolide inhibits NF-κB p65 activation in BLM induced pulmonary fibrosis in mice. Twenty-one days after BLM injection with or without andrographolide treatments, mice were sacrificed, and their lungs removed. The lung tissues were subjected to western blotting analysis using anti-phospho-NF-κB p65 and anti-NF-κB p65. The levels of phospho-NF-κB p65 in each sample were measured as ratios of intensities of phospho-NF-κB p65 to total NF-κB p65 bands. Each bar represents the mean ± SD of 10 mice. *p < 0.01 compared with Control; ^#^*p* < 0.01 compared with BLM; ^†^*p* < 0.01 compared with BLM + Andro-L.

**Figure 7.**
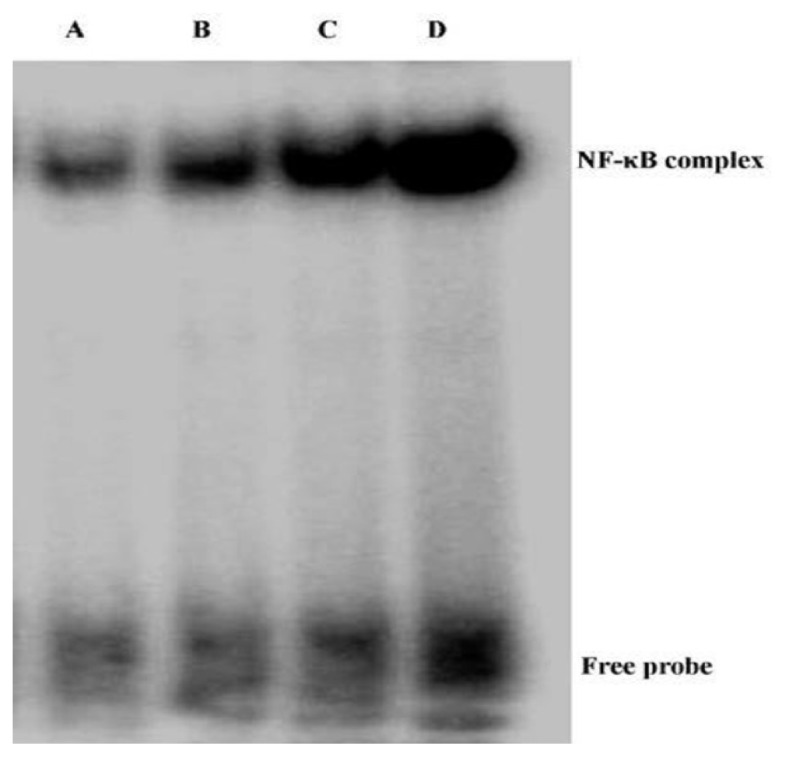
Andrographolide suppresses NF-κB p65 DNA binding activity in BLM induced pulmonary fibrosis in mice. Twenty-one days after BLM injection with or without andrographolide treatments, mice were sacrificed, their lungs were removed. NF-κB p65 DNA binding activity was examined by EMSA. (**A**) Control; (**B**) BLM + Andro-H; (**C**) BLM + Andro-L; and (**D**) BLM.

NF-κB, a wildly expressed and important transcription factor, contributed substantially to the regulation of BLM-induced inflammation and fibrosis [13–18]. Western blotting and EMSA were included to investigate the phosphorylation and DNA binding activity of NF-κB p65 in mice. The over activation of NF-κB p65 (phosphorylated NF-κB p65) was observed on day 21 after BLM injection. Meanwhile, our data also demonstrated that this noticeably increased p65 subunit phosphorylation was dose-dependently ameliorated by andrographolide (Figure 6). Furthermore, our EMSA findings revealed that NF-κB p65 DNA-binding activity was significantly increased on day 21 after BLM injection. However, the increased DNA-binding activity of NF-κB p65 was dose-dependently diminished by andrographolide administrations as well (Figure 7).

## 3. Discussion

Our present study found that andrographolide ameliorates bleomycin (BLM)-induced pulmonary inflammation and fibrosis probably through inactivation of NF-κB p65 in mice.

*Andrographis paniculata* (also named as the king of bitter), a traditional Chinese medicine, is wildly used in the treatment of inflammatory diseases, including asthma, pneumonia, viral infection, rheumatoid arthritis, fever, diarrhea and dysentery [11,19–21]. Recently, several studies have showed that the anti-inflammatory properties of andrographolide were resulted from inactivation of NF-κB at the level of the phosphorylation of IKKβ [11,19–21]. In the previous study, our data also showed that the severity of LPS-induced acute lung injury (ALI) was dose dependently ameliorated by andrographolide via inactivation of NF-κB in mice [11].

Idiopathic pulmonary fibrosis (IPF), a common pulmonary disease in the respiratory department, is a disorder associated with chronic persistent inflammation with activation of multiple immune cells, including macrophages and lymphocytes [1–6]. In spite of the many drugs and compounds that have been found and developed to treat IPF, the prognosis and mortality are still poor. The over 5-year survival rate is less than 50%, without lung transplantation [1–6]. BLM-induced pulmonary inflammation and fibrosis is the most common tool to explore and study the mechanism or test the therapeutic value of drugs in IPF [6–8].

Macrophages and lymphocytes contributed substantially to BLM-induced pulmonary inflammation and fibrosis [23–25]. After BLM injection, these inflammatory cells were activated, migrating into the inflammatory foci, synthesizing, and secreting various cytokines, inflammatory mediators, proteases, and reactive oxygen species (ROS) [23–25]; these active mediators could result in aberrant fibro-proliferation and collagen production in the lung tissues. It has been found that andrographolide was capable of suppressing the activation and migration of macrophages in a variety of inflammatory conditions [11,19–21,26]. Also, Iruretagoyena *et al*. indicated that allogenic-stimulated T cell activation was blocked by andrographolide [27]. Likewise, our data showed that total cells, macrophages, and lymphocytes in BALF were significantly raised on day 21 after BLM injection as well. Consistent with the previous data [11], our results also found that andrographolide reduced these cells counts in BALF in a dose-dependent manner. Additionally, a number of studies proved that TNF-α was critical in the pathogenesis of pulmonary fibrosis, leading to pulmonary fibrosis via TGF-β1 production [15,28,29]. Anti-TNF-α treatments demonstrated the beneficial effects on BLM-induced pulmonary inflammation and fibrosis, both *in vivo* and *in vitro* [30–32]. Our findings support that BLM up-regulation of TNF-α in BALF was dose-dependently compromised by andrographolide administration. Additionally, histological evaluation of lung sections 21 days after the BLM injection revealed evidence of notable inflammatory cells infiltration, alveolar wall and interalveolar septal thickening with excessive deposition of mature collagen. However, after andrographolide administrations, the pathological alterations in the lung tissues were relieved. Furthermore, in the current study, collagen deposition was determined by evaluating total hydroxyproline content of the lung tissues. Simultaneously, the Aschcroft score was included to assess and quantitate the pathological changes of the lung. Our data found that andographolide attenuated BLM-induced increment in hydroxyproline content and diminished Ashcroft score in a dose-dependent manner. These results indicated that andrographolide was capable of inhibiting BLM-induced pulmonary inflammation and fibrosis in mice.

Data from clinical studies and animal models revealed that TGF-β1 is a pivotal mediator with a wild spectrum of activities in inflammation, tissue repair, and fibrosis [28–32]. The up-regulation of TGF-β1 plays a critical role in the pathogenesis of BLM-induced pulmonary fibrosis [28–32]. Our findings demonstrate that TGF-β1 expression in lung tissues was noticeably elevated 21 days after BLM injection, which was consistent with previous reports [6,15]. Nevertheless, the overexpressed TGF-β1 was dose-dependently inhibited by andrographolide administrations. Additionally, EMT contributed substantially to the fibrotic response and α-SMA was considered to be a feature marker of myofibroblasts [17,18,27–32]. Moreover, Jiang *et al*. found that TGF-β1 is central to pulmonary fibrosis and could regulate the differentiation of pulmonary fibroblasts into myofibroblasts, which is characterized by α-SMA expression and active synthesis of extracellular matrix (ECM) molecules [15]. In our study, andrographolide down-regulated α-SMA mRNA and protein expression in BLM-induced lungs. These findings suggested that the anti-fibrosis effect of andrographolide probably resulted from the inhibition of EMT.

In IPF patients and animal models of pulmonary fibrosis, a significant and sustained inflammatory response characterized by the infiltrations of inflammatory cells were observed and the increased expression of inflammatory mediators, including TNF-α, TGF-β1, IL-1, inducible nitric oxide synthase (iNOS), and interferon (IFN)-γ were detected [27–32]. Regulation of these inflammatory mediators may be dependent upon the activation of NF-κB, due to their promoter regions containing binding sites for this transcription factor [11–13,18,20,21]. Unsurprisingly, various therapeutic strategies targeted at the NF-κB signaling pathway, including NF-κB–specific decoy oligonucleotide, p65-specific antisense oligonucleotide, and IKKβ-selective small molecule inhibitor have suggested positive effects in the pulmonary fibrosis and pulmonary inflammation associated disorders, including IPF, chronic obstructive pulmonary disease (COPD), acute lung injury/acute respiratory distress syndrome (ALI/ARDS), and asthma [20,33]. Our previous study found that andrographolide was a potent inhibitor of the NF-κB signaling pathway by inactivation of IKKβ, which is responsible for regulating the phosphorylation and nuclear translocation of NF-κB, in LPS-induced ALI [11]. According to our data, the BLM-enhanced phosphorylation level of NF-κB p65 and NF-κB p65 DNA binding activity were dose-dependently suppressed by andrographolide administrations. Therefore, we assumed that the anti-inflammatory and anti-fibrosis property of andrographolide is probably resulting from inactivation of the NF-κB signaling pathway.

Andrographolide has been widely used in China for a long period without any serious side effects or adverse events [11,20,21]. Thus, it has more advantages than a newfound or recently synthesized compound, which needs a very long time and heavy cost to test and evaluate for safety in clinical use. Nevertheless, clinical studies, particularly randomized-controlled trials (RCTs), should be carried out to further explore the therapeutic value of andrographolide in IPF.

## 4. Materials and Methods

### 4.1. Animals

All animal use procedures were approved by the Committee on the Ethics of Animal Experiments of West China Medical School, Sichuan University (Chengdu, China). This study was carried out in strict accordance with the recommendations in the Guide for the Care and Use of Laboratory Animals of the National Institutes of Health. All operations were performed under sodium pentobarbital anesthesia, and all efforts were made to minimize suffering. Six to eight weeks old specific pathogen-free male C57BL/6 mice (18–22 g) were obtained from the animal center in our university, and maintained under specific pathogen-free conditions. The mice were housed in a temperature-controlled room with 12 h dark/light cycles, and allowed food and water *ad libitum*. Animals underwent an acclimatization period of at least 1 week before use in our study.

### 4.2. Murine Model of BLM-Induced Pulmonary Fibrosis

Forty male C57BL/6 mice were randomly divided into 4 groups (*n* = 10), control group, BLM group, low dosage andrographolide (Sigma, St. Louis, MO, USA) treatment group (1 mg/kg, BLM + Andro-L group) and high dosage andrographolide treatment group (10 mg/kg, BLM + Andro-H group). According to the report, pulmonary fibrosis was induced by a single dose of BLM (3 mg/kg in 50 μL sterile saline, Sigma, St. Louis, MO, USA) via intratracheal injection [34]. In brief, mice were anesthetized with pentobarbital sodium (30 mg/kg), followed by BLM intratracheal injection with a 24-gauge needle. The mice in control group were administrated the sterile saline instead. Then, the mice were placed in a vertical position and rotated for 1 min to distribute the instillation in the lungs [11]. Ten minutes after BLM injection, andrographolide was given by intraperitoneal injection at 1 mg/kg in 100 μL PBS (BLM + Andro-L group) or 10 mg/kg in 100 μL PBS (BLM + Andro-H group). The next 20 days, the mice in the treatment groups received andrographolide injections per 12 h.

### 4.3. Bronchoalveolar Lavage Fluid (BALF) and Cell Counting

On day 21, mice were exsanguinated after pentobarbitone (50 mg/kg i.p. (intraperitoneal injection)) anesthesia. According to our previous report, BALF was collected [11,35]. In brief, BALF was obtained by cannulating the upper part of the trachea, by lavage twice with 1.0 mL PBS (pH 7.2). The fluid recovery rate was more than 85%. Lavaged sample from each mouse was kept on ice. BALF was centrifuged at 700× *g* for 5 min at 4 °C. The sediment cells were resuspended in 50 μL PBS and stained with Diff-Quik (International Reagents Corp., Hyogo, Japan) for cytospin preparations. Then, total cells, neutrophils, macrophages and lymphocytes were counted in a double-blind manner with a hemocytometer (Hausser Scientific, Horsham, PA, USA).

### 4.4. TNF-α in BALF

According to our previous study, the level of TNF-α in BALF was measured [6]. Briefly, the BALF supernatant was collected after centrifugation (for 4 min at 4000 rpm) and stored at −70 °C before the cytokine assay. TNF-α in BALF were measured by ELISA (R & D Systems, Minneapolis, MN, USA). The limit of detection of this method was better than 7.8 pg/mL.

### 4.5. Histopathological Examination

As described in our previous study, the right lower lung from each mouse was fixed in 10% formalin, embedded in paraffin, cut into 5 μm sections, stained with H & E [11,36]. Then, Masson’s trichrome (MT) staining was included in our study to observe the collagen deposition. Pulmonary pathological changes were observed and assessed by a blinded pathologist with Ashcroft score system [6].

### 4.6. Hydroxyproline Assay

According to our previous study, the total collagen content of the left lung tissues were analyzed by colorimetric assay to quantify hydroxyproline content in the lung tissues on day 21 after BLM injection [6]. Briefly, the minced left lung were homogenized in 6 mol/L HCl and hydrolyzed for 5 h at 130 °C. The pH was adjusted to 6.5–7.0 with NaOH, and the sample volume was adjusted to 30 mL with distilled water. The sample solution (1.0 mL) was mixed with 1.0 mL of chloramine T solution (0.05 mol/L), and then the mixture was incubated for 20 min at room temperature (RT). Then, 1.0 mL dimethyl benzaldehyde solution (20%) was added, the mixture was incubated at 60 °C for 20 min. The absorbance at 550 nm was analyzed. The result was demonstrated as μg hydroxyproline per mg wet lung (μg/left lung) by hydroxyproline standards (Sigma, St. Louis, MO, USA).

### 4.7. Reverse Transcription-Polymerase Chain Reaction (RT-PCR)

As described before, RT-PCR was used in our study to determine the mRNA expression of TGF-β1 and α-SMA in lung [11,37]. In brief, firstly, the right upper lung tissues were kept in −80 °C. Then, total RNA was isolated from the lung tissues by Trizol reagent (Invitrogen, Carlsbad, CA, USA). PCR was performed with a DNA thermal cycler in a 50 μL reaction volume, containing 5 μL 10× Taq Buffer, 4 μL 2.5 mM dNTP, 4 μL 25 mM MgCl2, forward and backward primers 2 μL each, Taq polymerase 0.5 μL and cDNA template 2 μL, for 35 cycles via GeneAmp PCR system 9700 (Applied Biosystems, Foster City, CA, USA). The primer sequences were as follows: *TGF-β1* (forward) 5′-CAACAAC GCCATC TATGAGA-3′ and (reverse) 5′-TATTC CGTCTCCT TGGTTC-3′; *α-SMA* (forward) 5′-AAGAG CATCCGACA CTGCTG-3′ and (reverse) 5′-AATAGCCA CGCTCAG TCAGG-3′; *β-actin*, (forward) 5′-CGAGCGGGCTACAGCTTC-3′ and (reverse) 5′-GTCACGCACGATTCCCTCT-3′. The mouse *β-actin* housekeeping gene was used as an internal control.

### 4.8. Western Blotting

As described before, the protein expression was determined by western blotting [35,37]. According to our previous report, the right middle lung tissues were kept in −80 °C. By the aid of a tissue grinder, the lung tissues were homogenized in PBS containing the protease inhibitor cocktail. The homogenates were centrifuged for 15 min at 14,000 rpm in 4 °C. Supernatants of lung tissues were collected, and protein concentration of each sample was measured with a bicinchoninic acid assay kit using BSA as standard (Pierce, Rockford, IL, USA). An equal amount of protein from each sample (150 μg) was resolved in 10% Tris-glycine SDS polyacrylamide gel. Protein bands were blotted to nitrocellulose membranes. After incubation for 1 h in blocking solution (5% dry milk in Tris-buffered saline with Tween 20) at room temperature (RT), the membrane was incubated for 24 h with anti-TGF-β1 (1:500 Santa Cruz Biotechnology, Inc., Santa Cruz, CA, USA), anti-α-SMA (1:800 Santa Cruz Biotechnology, Inc., Santa Cruz, CA, USA), anti-NF-κB p65 (1:800 Santa Cruz Biotechnology, Inc., Santa Cruz, CA, USA) or anti-phospho-NF-κB p65 (1:800 Santa Cruz Biotechnology, Inc., Santa Cruz, CA, USA), at 4 °C, respectively. The secondary antibody (horseradish peroxidase-conjugated donkey anti-rabbit immunoglobulin) was added at 1:10,000 dilution and incubated at room temperature for 1 h. Peroxidase labeling was detected with the enhanced chemiluminescence Western blotting detection system (Amersham Pharmacia Biotech, Piscataway, NJ, USA) and analyzed by a densitometry system. Mouse anti-β-actin antibody (1:5000 dilution; Santa Cruz Biotechnology, Inc., Santa Cruz, CA, USA) was used as housekeeping gene to confirm that the same amount of each protein was loaded.

### 4.9. Electrophoretic Mobility Shift Assay (EMSA)

EMSA was performed according to the manufacturer’s instructions. Briefly, the oligonucleotide probe (5′-CATCGGAAATTTCCGGAAATTTCCGGAAATTTCCGGC-3′/5′-GCCGGAAATTTCTG GAAATTTCCGGAAATTTCCGATG-3′) was applied. Nuclear proteins were prepared as previously described [38]. In brief, nuclei were isolated, and nuclear extracts were prepared from the lung tissues by a Nuclear Extract Kit (Activemotif, Carlsbad, CA, USA). Binding reactions were performed using a LightShift Chemoluminescent EMSA Kit (Pierce Biotechnology, Rockford, IL, USA). The reaction mixtures (10 μL) containing about 5 μg nuclear extracts and 2 pmol of oligonucleotide probe were incubated for 20 min at RT. Specific binding was confirmed by a 200-fold excess of unlabeled probe used as a specific competitor. Protein DNA complexes were separated on a 6% non-denaturing acrylamide gel, transferred to positively charged nylon membranes, and cross-linked using a Stratagene crosslinker (Stratagene, La Jolla, CA, USA). Band shift was visualized with a streptavidin-horseradish peroxidase through chemoluminescent detection.

### 4.10. Statistical Analysis

Statistical analyses were performed with Sigmaplot software (SPSS) version 17.0 software (IBM Corporation, Somers, NY, USA). Each point corresponds to mean ± SD Statistical differences were determined by two-way analysis of variance (ANOVA) and *p* < 0.05 was considered to be statistically significant.

## 5. Conclusions

Our data demonstrate that andrographolide reduced the severity of BLM-induced pulmonary fibrosis probably through inactivation of the NF-κB signaling pathway. These data suggest andrographolide may be considered as an effective and safe drug for the potential treatment of IPF. Nevertheless, animal studies are limited to hours while IPF patients often are treated for several months or years. Taken together, we believe that andrograophlide may exert its beneficial effects over time when it is given at the early stage of IPF.
